# Subaerial weathering drove stabilization of continents

**DOI:** 10.1038/s41586-024-07307-1

**Published:** 2024-05-08

**Authors:** Jesse R. Reimink, Andrew J. Smye

**Affiliations:** Department of Geosciences, Pennsylvania State University, University Park, PA USA

**Keywords:** Geochemistry, Precambrian geology, Geodynamics

## Abstract

Earth’s silica-rich continental crust is unique among the terrestrial planets and is critical for planetary habitability. Cratons represent the most imperishable continental fragments and form about 50% of the continental crust of the Earth, yet the mechanisms responsible for craton stabilization remain enigmatic^1^. Large tracts of strongly differentiated crust formed between 3 and 2.5 billion years ago, during the late Mesoarchaean and Neoarchaean time periods^2^. This crust contains abundant granitoid rocks with elevated concentrations of U, Th and K; the formation of these igneous rocks represents the final stage of stabilization of the continental crust^2,3^. Here, we show that subaerial weathering, triggered by the emergence of continental landmasses above sea level, facilitated intracrustal melting and the generation of peraluminous granitoid magmas. This resulted in reorganization of the compositional architecture of continental crust in the Neoarchaean period. Subaerial weathering concentrated heat-producing elements into terrigenous sediments that were incorporated into the deep crust, where they drove crustal melting and the chemical stratification required to stabilize the cratonic lithosphere. The chain of causality between subaerial weathering and the final differentiation of Earth’s crust implies that craton stabilization was an inevitable consequence of continental emergence. Generation of sedimentary rocks enriched in heat-producing elements, at a time in the history of the Earth when the rate of radiogenic heat production was on average twice the present-day rate, resolves a long-standing question of why many cratons were stabilized in the Neoarchaean period.

## Abstract (web summary)

The geological histories of Archaean regions indicate that stabilization of the Earth’s continents and the formation of cratons was driven by continental emergence and subaerial weathering.

## Main

The most enduring blocks of continental crust, cratons, form refractory nuclei that have remained stable for billions of years and are some of the longest-lived and expansive geological features on Earth. Archaean cratons (arising more than 2.5 billion year ago (Ga)) host most of the global gold and platinum inventories and are important repositories of other critical mineral deposits, such as lithium-bearing pegmatites. Cratons also contain key archives of ancient planetary environments including the oldest preserved rocks of the Earth^4^, as well as records of ancient surface conditions^5^ and the climatic response to changes in the solid Earth system^6^. Cratons, defined here as blocks of more than 150-km-thick stable lithosphere^1^, are preserved in the Archaean rock record as Eoarchaean to Neoarchaean (4.0–2.5 Ga) granitic intrusions and supracrustal rocks that comprise Archaean ‘granite-greenstone’ belts. They have exceptional longevity—these packages of crust have remained stable and isolated from tectonic reworking for billions of years^7^. How these unique lithospheric domains were stabilized remains unresolved.

**Fig. 1:**
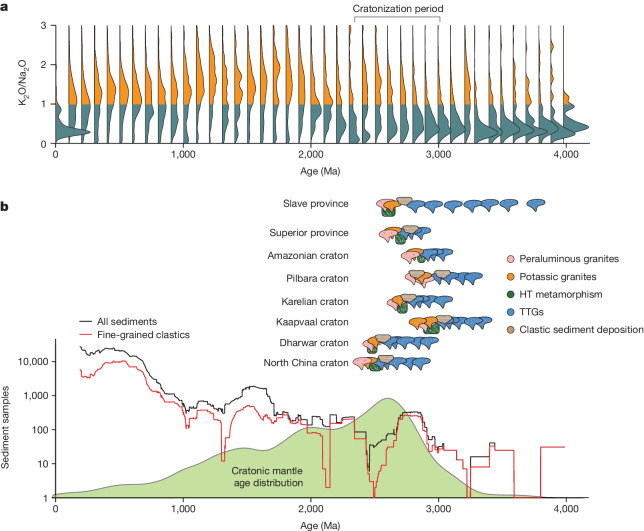
Geological evolution of cratons at the end of the Archaean. **a**, A stacked kernel density estimator for the composition of felsic (more than 62 wt% SiO_2_) igneous rocks through time, discretized in 50 Myr age bins. The total area under each probability density distribution is equal to 1, and the bottom of each density distribution is positioned at the minimum age of the given age bin that was used to subset the whole-rock geochemical database. Rocks with K_2_O/Na_2_O > 1 are rare before 3.0 Ga and common after 3.0 Ga. **b**, A compilation of granitoid geochemistry from various cratons^2^. Sedimentation, high-temperature metamorphism and emplacement of Neoarchaean granites occurred at different times in each craton. Data sources are presented in the Extended Data. The lowermost green field in **b** shows the normalized cratonic mantle age distribution^1^, with a prominent peak in the Neoarchaean period. Red and black lines show the number of preserved sedimentary (black) and fine-grained (red) rocks samples in a global database averaged in a moving-window calculation. Sedimentary rocks first appear en masse in the Mesoarchaean to Neoarchaean.

Critical to craton stability is the enrichment of the heat-producing elements (HPEs), uranium (U), thorium (Th) and potassium (K), in the upper crust relative to the lower crust. This serves to reduce temperatures in the deep crust and uppermost mantle, thereby strengthening the lithosphere to the extent that it becomes resistant to deformation^8,9^. Intracrustal transport of the HPEs occurs predominantly during melting, typified by postorogenic magmatism, whereby partial melting concentrates HPEs in the melt phase, which ascends to depths of neutral buoyancy in the crustal column. Thus, from a crustal perspective, craton formation is marked by the timing of emplacement of postorogenic granitoids, sometimes referred to as the Neoarchaean granite bloom^7,10^. The timing of this key crustal differentiation event differs between cratons but consistently occurs between about 3.1 and 2.5 Ga (Fig. 1b; refs. ^2,11^). The granite bloom was succeeded by tectonic quiescence over timescales of hundreds of millions to billions of years, indicating that this process represents the final stage of cratonization^11^. Importantly, these plutonic suites contain the first widespread evidence for potassium-rich granites; Mesoarchaean and older granitoids are dominated by distinctly different rock compositions^12,13^. Pregranite-bloom rocks contain mostly sodic (K_2_O/Na_2_O less than about 0.7) tonalite-trondhjemite-granodiorite-suite rocks (TTGs) and associated mafic rocks, forming Archaean ‘grey gneiss’ provinces^13^. These TTG-suite rocks are widely believed to be formed by partial melting of basaltic protocrust^13,14^ and thus represent primary additions to the felsic continental crust, not the products of final stabilization.

**Fig. 2:**
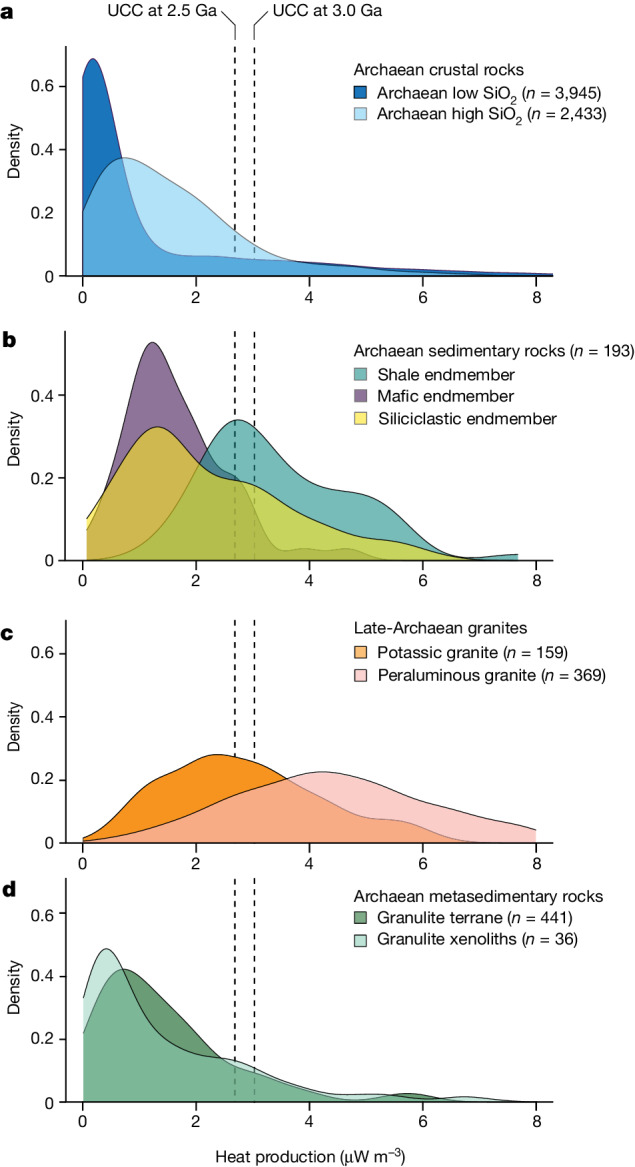
Heat production rates for Archaean rocks. All values are back-calculated to 2.8 Ga using measured concentrations of U, Th and K; each curve is a kernel density estimator for the underlying datasets. Vertical lines correspond to heat production of modern upper crust^22^ (UCC) calculated at 2.5 and 3 Ga. Source data and calculations are detailed in the Methods. **a**, Heat production rates for Archaean crustal rocks with silica content greater than (Archaean high-SiO_2_) or less than (Archaean low-SiO_2_) 62 wt%. **b**, Compositional endmembers of Archaean sedimentary rocks. **c**, Late Archaean granites broken apart into compositional categories. **d**, Archaean metasedimentary rocks exposed as granulite terranes and xenoliths.

By contrast to the older TTG-suite rocks, Neoarchaean granites are potassic (K_2_O more than 2.0%, K_2_O/Na_2_O more than about 0.7; Fig. 1a) and can be strongly peraluminous (herein defined as granites with molar Al/(2Ca − 1.67P + Na + K) > 1.1), and their chemical characteristics are incompatible with partial melting of mafic protocrust. Instead, the genesis of potassic and peraluminous granites require melting of older, intermediate composition continental crust (TTG crust) and sedimentary protoliths, respectively^2,15,16^. Thus, Neoarchaean granites represent the final stage of crustal differentiation in the formation of the continents on Earth. Neoarchaean granites are enriched in the HPEs U, Th and K (Fig. 2c) such that their formation by partial melting substantially depleted the lower crust of these elements, serving to strengthen and stabilize lithospheric blocks.

Prevailing explanations for the petrogenesis of the Neoarchaean granites invoke heating of the lithosphere to induce partial melting of pre-existing crust. This requirement is supported by Neoarchaean metamorphic terranes that preserve a record of high-temperatures in the middle and lower crust over timescales consistent with radiogenic heating in thickened crust^17,18^. Previously proposed mechanisms include mantle plumes impinging on the base of the lithosphere^19^, lithospheric inversion^17^, convective removal of lithospheric mantle^16^, conductive equilibration of thickened crust^2,20^ and advection of magmatic heat^21,22^. Although several aspects of these models are appealing, no one model can successfully account for all the geological constraints necessary to explain formation of cratonic nuclei in the Neoarchaean period.

A major obstacle, at least for some well-studied cratons, to models that invoke asthenospheric heat transfer as a mechanism to induce crustal melting is that formation of thick mantle lithosphere preceded (more than 2.8 Ga) granitoid emplacement in many cratons^1,23^. Impinging mantle plumes or convective instabilities would destroy such lithospheric roots^17^; the existence of older mantle roots thus renders asthenospheric heating an unlikely heat source for widespread crustal melting in some areas. By contrast, conductive equilibration of thickened lithosphere with radiogenic crust offers a satisfactory explanation for the attainment of supra-solidus crustal temperatures^24^ while maintaining thick lithosphere. Indeed, such thickening has been invoked to account for the thermal evolution of the thickest tract of lithosphere on modern Earth beneath Tibet^20^. In particular, it is important to note that granitoids with comparable compositions to the Neoarchaean granite-bloom event can be produced by this mechanism on the modern Earth^25^. However, the invocation of crustal thickening to explain widespread anatexis requires anomalous crustal enrichment of the HPEs relative to the composition of modern continental crust.

Here, we use the Archaean rock record to quantify the heat production rates of Archaean TTG terranes. Global compilations^26,27^ show that these terranes have substantially lower heat production (1–2 μW m^−3^ at 2.8 Ga; Fig. 2d) than modern crustal compositions would have had in the Mesoarchaean period (about 3–4 μW m^−3^). This finding implies that extrapolation of crustal thickening to the Mesoarchaean period based on modern crustal compositions^20^ overlooks notable secular changes in the composition of crust^12^ and is therefore not warranted. We build on previous efforts that have demonstrated the importance of radiogenic heating for the production of Archaean cratonic lithosphere^20,28^ by combining rock-specific calculations of Neoarchaean heat production with thermal models to evaluate the potential for thickening to drive crustal differentiation.

## Archaean heat production

To quantify the heat production rates of typical rock types found in Archaean terranes, we compiled geochemical bulk-rock analyses and calculated heat production rates at 2.8 Ga, an average age for the onset of granite-bloom events (Fig. 2), using present-day U, Th and K concentrations in each rock type. We used major element rock compositions to define two groups of Archaean crustal rock types, a high-Si group (more than 60 wt% SiO_2_) that is dominated by TTG-like compositions and a low-Si grouping (less than 60 wt% SiO_2_) that corresponds to basaltic rocks. Sedimentary rock data were partitioned into siliciclastic, shale and mafic sediment groups; our Archaean shale composition represents an estimate of an archetypal sediment composition used in the modeling. Details of the calculations are provided in the Methods.

In the Mesoarchaean period, radiogenic heat production was around double the modern rate, but this depends on the specific concentrations of each radioactive isotope (as a result of the dramatic differences in the half lives of ^40^K, ^232^Th, ^235^U and ^238^U). Common to all Archaean cratons is the predominance of TTG suites in polymetamorphosed basement gneiss complexes^13^. Our calculations show that by the Neoarchaean period, felsic igneous crust—dominated by sodium-rich, intermediate to felsic TTG granitoids—had heat production rates of less than 2 μW m^−3^ (the high-Si group: median = 1.28, s.d. = 1.76, 75th percentile = 2.2, *n* = 2,433; Fig. 2a). This defines the upper limit of the heat production capacity of pregranite-bloom Archaean crust as Archaean terranes contain a mix of TTG and mafic gneisses^7,29^—the addition of mafic rocks to this package will reduce the internal heat production capabilities of pregranite-bloom Archaean crust (the low-Si group: median = 1.13 μW m^−3^, s.d. = 2.6, 75th percentile = 1.16, *n* = 3,945). By stark contrast, Archaean sedimentary rocks from felsic sources (data from ref. ^30^), have significantly elevated heat production values that range from 1 to 6 μW m^−3^ (median = 2.54 μW m^−3^, s.d. = 1.5, 75th percentile = 3.66, *n* = 193; Fig. 2b) with an asymmetric distribution to higher values. Of these sediments, shales have the highest heat production rates (3–5 μW m^−3^) as they are enriched in U compared to non-shales. HPEs are also concentrated in both potassic (1–5 μW m^−3^) and peraluminous (2–8 μW m^−3^) Neoarchaean granites (data from refs. ^2,15,31^). Peraluminous granites necessitate sedimentary source rocks, while many potassic granites in the Neoarchaean have isotopic signatures that implicate involvement of older continental crust^2^. Further, as many melt/bulk-rock partition coefficients for the HPE are less than 1 (refs. ^32,33^), this implies that these peraluminous granites formed from HPE-enriched sedimentary protoliths. The potential for fractional crystallization to cause HPE-enrichment requires further evaluation; small grain-sizes of U- and Th-bearing accessory phases make their physical separation from the melt implausible until they become included by a crystallizing major mineral.

## Thermal evolution of thickened crust

Our assessment of Archaean heat productivity based on actual rock compositions allows us to investigate the role of radiogenic heat production in cratonization. We use these rock-based heat production values to constrain one-dimensional thermal models of the cratonization process in which the thermal structure of thickened crust evolves through the combined effects of conductive relaxation of isotherms and radiogenic heating following thickening. Details of the calculations are provided in the Methods. Critically, we examine the ability of various distributions of crustal heat production to cause partial melting and differentiation of the continental crust in the Neoarchaean period.

**Fig. 3:**
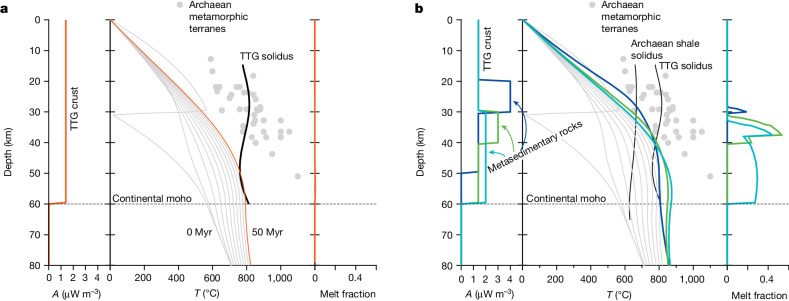
Thermal evolution of thickened crust in the Neoarchaean. **a**, Thermal evolution of thickened TTG crust. Left-hand plot shows the vertical distribution of heat production (*A*) for each thermal model; middle plot shows geotherms in 5 Myr increments following instantaneous thickening of 30-km-thick crust by a factor of two (‘saw tooth’ initial geotherm). The thick orange line represents the geotherm at 50 Myr, the solid black line shows the TTG solidus (‘dry’ melting of high-Si bulk composition) and the grey markers represent a compilation of *P–T* estimates derived from Archaean metamorphic terranes^59^. Melt fractions corresponding to the 50 Myr geotherm are shown in the panel farthest to the right. Note that this crustal configuration does not result in significant melt production (less than 2 vol% melt at 50 Myr). **b**, As for **a** except for distributions of heat production that correspond to sediment burial. Combinations of sediment layer thickness and heat production are chosen to demonstrate the importance of emplacement depth on thermal structure: lower thermal gradients with increasing depth mean that lower rates of heat production are required to cause melting. For the sedimentary layer, melt fractions were calculated using *T–X* relations derived for the Archaean shale composition (Extended Data Table 1). Coloured lines show geotherms calculated at 50 Myr for crustal configurations that contain radiogenic sedimentary layers placed at various depths and thicknesses (heat production shown on the left panel). Black lines correspond to solidus curves for TTG (as in **a**) and ‘wet’ shale composition. Abrupt changes in melt fractions with depth are caused by the exhaustion of muscovite during melting.

**Fig. 4:**
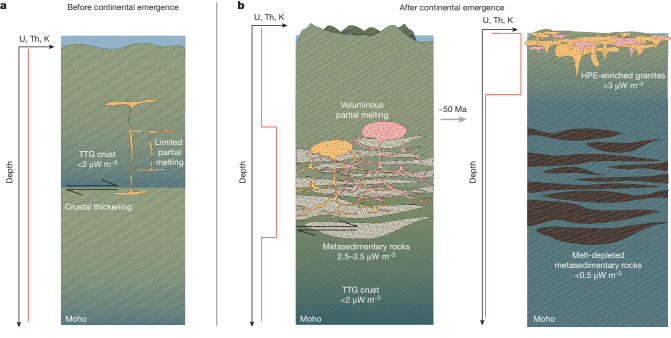
Subaerial weathering drives cratonization. Schematic illustration of the key geodynamic processes in craton stabilization. **a**, Before continental emergence, thickening of TTG crust did not cause extensive melting, resulting in only limited crustal differentiation. **b**, Following continental emergence, weathering of TTG crust concentrated U, Th and K into terrigenous sedimentary rocks, which, when entrained into the deep crust, elevated the crustal geotherm over tens of millions of years and induced large degrees of melting. Subsequent melt-migration redistributed the HPEs from the lower to the upper crust (potassic and peraluminous granites, yellow and pink, respectively), thermally and mechanically stabilizing the continental lithosphere. Vertical orange lines represent schematic distributions of U, Th and K.

Our calculations show that thickening of Archaean TTG crust for a range of geologically plausible parameters fails to result in significant partial melting. Figure 3a shows an example result for TTG crust (1.4 μW m^−3^, evenly distributed) in which 30-km-thick crust is instantaneously thickened by a factor of two. While this configuration results in more than 400 °C heating of the middle and lower crust over 50 Myr, the peak temperatures attained do not significantly exceed the TTG solidus (700–800 °C over the crustal pressure range, calculated using average TTG composition; Methods) and produce only low-volume melts (less than 2%) that are insufficient to account for the voluminous record of magmatism preserved in the Neoarchaean granitoid rock record^2,15,31^. This finding suggests that stacking of even pure-TTG crust before the Neoarchaean granite bloom could not produce the hallmark geological signatures of cratonization^20^ and that an alternative heat source is required to produce the voluminous granitoids that mark the stabilization of Archaean continental nuclei globally (Fig. 4).

The common occurrence of metasedimentary rocks in Mesoarchaean and Neoarchaean granulite terranes^34,35^ implies that heat production in thickened Neoarchaean crust exceeded that of pure-TTG crust. Our calculations show that the incorporation of sediments into thickened crust has the potential to induce significant melting of both the sediment and adjacent TTG crust. Figure 3b shows the effect of sediment incorporation on the thermal evolution of Mesoarchaean crust; for example, a 10-km-thick layer of sedimentary rock with an average heat production of 3 μW m^−3^ incorporated at 30 km depth results in granulite-facies metamorphism and the generation of granitic melt fractions exceeding 40% after 50 Myr (green lines, Fig. 3b). Elevated rates of heat flow through the upper crust mean that the potential for sediments to undergo partial melting increases with depth of burial such that the distributed incorporation of low-HPE sediments throughout the deep crust (2 μW m^−3^ between 30 and 60 km) is sufficient to yield melt volumes that locally exceed 25 vol% (for example, light blue line, Fig. 3b). The heat generated from buried sediments also stimulates partial melting of adjacent, non-metasedimentary crustal lithologies (Extended Data Figs. 9 and 10), producing cogenetic suites of metaluminous and peraluminous granitoids. A further consequence of deep sediment burial and focused radiogenic heating is the downward conduction of heat from the lower crust into the underlying mantle lithosphere. Provided the negative heat flux is sufficient to cause melting of fusible rocks in the uppermost mantle lithosphere, this mechanism could account for the occurrence of minor volumes of mantle-derived mafic rocks exposed in some cratons during this interval.

## Geology of Archaean cratons

Our calculations imply that the onset of terrigenous sedimentation in the Mesoarchaean period, caused by the increase in continental freeboard^5^, resulted in a step change in the ability of the crust to undergo internal chemical differentiation. Weathering of TTG crust concentrated HPEs into sedimentary lithologies whose burial provided the heat source required to internally differentiate the continental crust and produce cratons.

Large volumes of sedimentary rocks have not been present on the surface of the Earth throughout the rock record. In fact, mature sedimentary packages are only preserved on Earth since the Mesoarchaean (Fig. 1b)^5,36^. The Archaean felsic TTG crust has long been known to have been submerged beneath sea level—as shown in large part by submarine basaltic packages that overlie most Mesoarchaean basement gneiss assemblages (for example, ref. ^7^). Although sedimentary packages are known to occur back to 3.8 Ga, before around 3.0 Ga they tend to consist of thin, immature, ‘cover-group’ sequences that have restricted catchments and probable volcanogenic origins^37^. During the Mesoarchaean, large sedimentary basins, including fine-grained sediments, were deposited on pre-existing continental crust. For instance, the Witwatersrand basin, deposited at ~2.7 Ga, contains up to 6 km of interbedded clastic sequences^38^; Mesoarchaean and Neoarchaean sedimentary basins indicative of exposed continental crust exist in many other cratons, including the Slave (up to 5 km of turbidites and shales at 2.67–2.63 Ga; ref. ^39^), Pilbara (more than 2.9 Ga, including the De Gray Superbasin^40^) and Kaapvaal craton (the 3.26 Ga Fig Tree Group containing up to 1 km of shale^41^). Importantly, large volumes of HPE-enriched sedimentary rocks are not found on Earth before about 3.0 Ga (Fig. 1b) despite the fact that there are large tracts of pre-3.0 Ga crust found in Archaean ‘grey gneiss’ terranes—such terranes are dominated by orthogneisses of igneous origin.

Our proposed mechanism makes specific predictions for the relative timings of sedimentation, metamorphism and magmatism in the Neoarchaean period. Sedimentation is required to be antecedent to metamorphism and crustal melting and we logically expect that both Neoarchaean K-rich granitoids and exhumed granulite-facies metasedimentary rocks are consanguineous. Furthermore, granulite-facies metamorphism in the middle and lower crust should precede or be contemporaneous with the timing of plutonism in a specific cratonic region. There is ample geochronological and petrological evidence from specific regions that supports these predictions (Fig. 1b and Methods). For instance, the Neoarchaean granite-bloom event, comprising both peraluminous and potassic granitoids (Extended Data Fig. 8 and Methods) of the Slave craton occurred between 2.62 and 2.58 Ga (ref. ^21^) but is predated by clastic sediment deposition (2.69–2.66 Ga; ref. ^7^), whereas metasedimentary xenoliths preserve a record of granulite-facies metamorphic conditions at around 2.62–2.59 Ga (ref. ^42^), correlating with granitoid emplacement. These granulite-facies Archaean metasedimentary rocks are depleted in HPE and underwent partial melting at lower-crustal conditions^34^. Lower crust in the Kaapvaal craton, exposed by the Vredefort impact event, contains metasedimentary rocks that underwent granulite-facies metamorphism at around 3.0 Ga (ref. ^43^), succeeded by voluminous granitoid plutonism^2^. Moreover, combined zircon O- and Hf-isotope systematics from Kaapvaal peraluminous granites imply a negligible influence of mantle-derived melts during sediment melting under lower-crustal conditions, consistent with granite formation in response to radiogenic heating from HPE-enriched sediments^44^. Granite formation was coeval with metamorphism in the deep crust of the Kaapvaal^35^ and resulted in the redistribution of HPEs to the near-surface and net strengthening of the crust^3^.

At a global scale, our model predicts that granulite-facies metamorphism of sedimentary protoliths occurred during or before Neoarchaean cratonization and that large tracts of melt-depleted metasedimentary crust now exist at depth in Mesoarchaean and Neoarchaean orogenic crust. This requirement finds support from the observation that about 45% of samples from exhumed Archaean granulite terranes have peraluminous compositions^45^, consistent with a metasedimentary origin and the incorporation of near-surface rocks into the Neoarchaean lower crust. Furthermore, the conditions of metamorphism preserved by these rocks imply heating in middle and lower orogenic crust^46^ (see pressure–temperature (*P–T*) data shown in Fig. 3). The few constraints that exist on the pressure–temperature–time evolution of such metamorphism indicate that granulite-facies metamorphism occurred in thickened crust^47^ at a similar time to the Neoarchaean granite-bloom emplacement (Fig. 1b). Slow seismic wavespeeds and low *V*_P_/*V*_S_ ratios measured through some Archaean cratonic regions (for example, Kaapvaal craton^48^) provide more support for the contention that portions of deep cratonic crust contain significant volumes of metamorphosed sedimentary rocks.

One aspect of the geological record that may seem to be at odds with our model is the observation that peraluminous granites are subordinate to potassic granitoids on some Neoarchaean cratons^15^. The mechanism presented here predicts that melting was primarily driven by sedimentary heat production; however, it does not necessarily suggest that the Neoarchaean magmatic signature should be dominated by pure sediment melts as the incorporation of sediments into the deep crust can stimulate the production of significant proportions of melt from pre-existing crust (Methods). Generation of melts from pre-existing TTG-like crust draws strong support from Nd- and Hf-isotope systematics of the global array of Neoarchaean granites^2^, notably including a large proportion of the western Slave craton^21^. Furthermore, post-Archaean erosion may have removed upper crustal material containing such melts, as inferred from zircon O-isotope ratios^49^.

## Tectonic style need not have changed

Our proposed mechanism for cratonization, whereby weathering of emergent continents concentrated HPE into sediments that drove intracrustal differentiation, does not require a global reconfiguration in tectonic style in the Neoarchaean period to explain the observed increase in lithological diversity across this time period^12^. The mechanism is also consistent with the existence of cratonic mantle before Neoarchaean granite formation, as probably required for continental emergence^1^. Several lines of evidence indicate that such lithospheric mantle was stabilized by imbrication^23^, generating tracts of continental crust that could maintain freeboard and produce expansive sedimentary basins in the Mesoarchaean–Neoarchaean. These HPE-enriched sedimentary rocks were then incorporated into the deep crust by compressional tectonic activity that resulted in widespread melting and plutonism and was associated with the genesis of high-K, ‘sanukitoid’ magmatism produced by mantle-wedge melting^50^. Incorporation of sedimentary rocks into the deep crust caused widespread crustal melting and plutonism; melt-migration redistributed HPE from the middle and lower crusts to the upper crust, which, in turn, drove cooling and strengthening of cratonic crust. Although Mesoarchaean TTG-dominated crust was incapable of undergoing such differentiation, the appearance of rocks that concentrate HPEs in a plate tectonic regime is sufficient to cause intracrustal differentiation.

We emphasize that this mechanism has no specific requirement for the physical process by which sediments are incorporated into the deep crust. This may have occurred by relamination^51^, tectonic underplating^52^ or burial^53^; such processes are active on modern Earth and probably occurred in the Neoarchaean period^2^. Contrary to previous interpretations, we infer that tectonic regime need not have changed from the Mesoarchaean to Neoarchaean period to account for the geological evolution of Archaean cratons. Instead, the mechanism proposed implies that craton stabilization was activated by continental emergence, which, in turn, was driven by one of several viable processes unrelated to tectonic transitions^54–58^. The geological record can then be cast in terms of a pre-emergence (TTG-dominated) and post-emergence (granite-dominated) planet. These findings demonstrate the importance of exogenic processes for the geodynamic evolution of planetary interiors and the generation and sustenance of habitable conditions. Ultimately, the onset of planetary-scale subaerial weathering led to not only dramatic atmospheric change but also drove final distillation and stabilization of continents.

## Methods

### Whole-rock heat production calculations

For all samples, the modern U, Th and K concentrations were used to calculate total rock heat production back in time. Each radioactive isotope (^40^K, ^232^Th, ^235^U and ^238^U), along with their respective decay constants, was used to calculate the heat production of each rock. This type of sample-based calculation is essential to obtain a clear picture of the heat production in Archaean rock compositions, as the distributions of U, Th and, in particular, K, vary between rocks and over time. For consistency, we calculated the heat-producing capabilities of each rock sample at 2.8 Ga, then compiled these values (Fig. 2a–d).

To determine the sample-based heat production of Archaean crustal rocks and sediments, we compiled modern (that is, measured) concentrations of U, Th, K (in ppm) and major elements (in wt% oxide; SiO_2_, CaO, TiO_2_, Al_2_O_3_, FeO_T_, MgO, Na_2_O, K_2_O, in which FeO_T_ corresponds to total Fe) from published whole-rock compilations. The major element composition of each sample was used to define a statistical framework that enables us to compute average and median values of heat production for specific lithologies.

**Extended Data Fig. 1:**
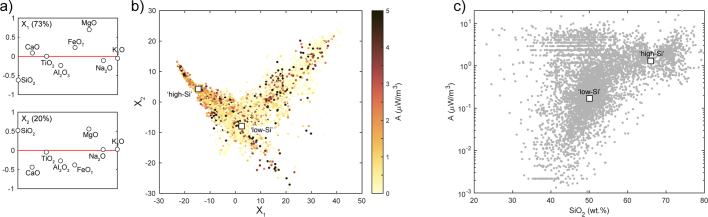
Principal components analysis (PCA) of Archean crustal rocks. Panel **a** shows principal component loads for Archean igneous and metamorphic rocks (referred to as ‘crustal rocks’ throughout). The first principal component (X_1_) dominates the compositional variance (73%) and separates silica-rich from mafic compositions; the second principal component accounts for a subordinate proportion of the total variance (20%) and separates compositions enriched in CaO, Al_2_O_3_ and FeO_T_ from those enriched in SiO_2_ and MgO. Panel **b** shows principal component scores for the compiled dataset of 6691 whole-rock analyses shaded according to radiogenic heat production at 2.8 Ga. Square markers correspond to exemplar ‘high-Si’ and ‘low-Si’ compositions defined in panel **c** that, in turn, shows heat production calculated at 2.8 Ga plotted against bulk-rock SiO_2_ wt.%.

For Archaean crustal rocks, we compiled whole-rock compositions from two sources (Fig. 2): compositions of Archaean TTG ‘grey gneisses’ from ref. ^29^ were combined with a large compilation of Archaean crustal rock compositions from ref. ^60^ (file ‘aad5513-tang.m-sm.database.s2.xlsx’ from their supplementary material). The conjoined dataset was filtered to remove partial analyses (those that did not report values for U, Th or K or the major elements), reducing the sample set to 6,691 samples for which we calculate heat production values. A principal components analysis (PCA) of non-normalized major element data shows that most compositional variance (73%; Extended Data Fig. 1a,b) is accounted for by classical indices of magmatic differentiation (SiO_2_ and MgO concentrations). We use non-normalized data for the PCA, reflecting our interest in absolute—and not relative—values of compositional variance; all PCA calculations were performed using the ‘princomp’ function of MATLAB. Extended Data Fig. 1c shows that the aggregate dataset defines a broad positive correlation between heat production and whole-rock SiO_2_ concentration, as is expected for plutonic rocks^61^. The correlation defines an obvious grouping threshold of 60–65 wt% SiO_2_, separating high-SiO_2_ (*n* = 2,046) from low-SiO_2_ (4,645) compositions, which we use to compute population statistics for heat production (main text); the distributions defined by these data are plotted in Fig. 2. Exemplar major element compositions for each group are shown as square markers on Extended Data Fig. 1b,c and used in phase equilibria calculations detailed beneath.

**Extended Data Fig. 2:**
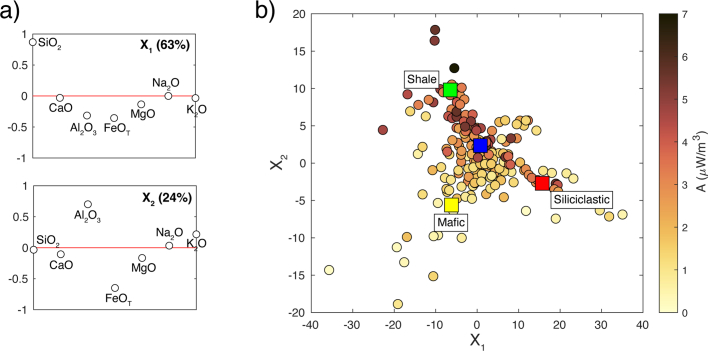
Principal components analysis of Archean sedimentary compositions. Panel **a** shows principal component loads calculated from non-normalized bulk-rock compositions. The first principal component (X_1_) accounts for 63% of the compositional variance and separates silica-rich sediments from other compositions; the second principal component (X_2_) accounts for 24% of the variance and separates aluminous sediments from more ferruginous compositions. Panel **b** shows PCA scores for 269 Archean sedimentary compositions shaded according to heat production calculated at 2.8 Ga. endmember compositions are shown by the red (siliciclastic), green (shale) and yellow (mafic sediment) square markers; the blue marker corresponds to our exemplar ‘Archean shale’ sediment composition used in the phase equilibria calculations.

Archaean sedimentary rock heat production values were determined using the database of compositions from ref. ^30^. This compilation was filtered for rock samples with a complete set of major element and U, Th and K concentrations, with depositional ages of more than 2.5 Ga (*n* = 269). We performed a PCA on the non-normalized dataset, finding that two axes account for more than 80% of the compositional variance. The first axis (*X*_1_; Extended Data Fig. 2a) separates SiO_2_-rich compositions (arenites) from those rich in FeO and Al_2_O_3_ (mafic sediments and shales); the second axis (*X*_2_) separates aluminous from ferruginous sediment compositions. These axes define three obvious endmembers: siliciclastic, shale and mafic sediment compositions, between which the aggregate data form mixing arrays. Bulk-rock heat production increases towards the shale endmember (Extended Data Fig. 2b). For our calculations of heat production, we partitioned data according to orthogonal distance from each endmember sediment composition (that is, which ‘arm’ of the *X*_1_ versus *X*_2_ structure the sample is aligned to). We then computed population statistics on the heat production for each of the three groupings, siliciclastic, shale and mafic sediments, respectively; Fig. 2 shows the distribution of these data.

Compositions of late Archaean granite samples were aggregated from three sources: (1) peraluminous granites^15^ (*n* = 84); (2) a Kaapvaal granite database^2^ (*n* = 51); and (3) a Slave craton granite database^16,21^ (*n* = 109). Filtering for analyses with U, Th and K_2_O measurements reduced the database from 160 samples to 159 granite samples from the suite of samples from the Slave and Kaapvaal and 84 peraluminous Archaean samples. Reported (modern) U, Th and K_2_O values were used to calculate heat production rates for each rock analysis at 2.8 Ga and these values are plotted in Fig. 2.

Heat production rates for Archaean lower-crustal rocks from exhumed granulite-facies terrains and xenolith suites were calculated in a similar manner using the compilation of ref. ^45^. Of these samples, 36 metasedimentary xenoliths had U, Th and K_2_O values, whereas 441 metasediments from Archaean granulite terrains had all three values allowing for an accurate calculation of the heat production at 2.8 Ga.

Heat production rates for modern continental crust back in time are based on the calculations of ref. ^20^ who used the composition of modern upper continental crust from ref. ^62^.

### Phase equilibrium calculations

The effect of the latent heat of melting on thermal evolution is dependent on the relationship between temperature (*T*), pressure (*P*) and melt fraction (*X*). As the equilibrium melt fraction at any given *P–T* condition is controlled by the availability and compositions of fusible minerals, we calculated *T*(*P*)–*X* relations for each of the compositions detailed above.

Phase relations were calculated using Theriak-Domino v.11.03.2020, downloaded from https://titan.minpet.unibas.ch/minpet/theriak/theruser.html (ref. ^63^) combined with the 2011 version of the Holland and Powell thermodynamic database (ds62, update February 2012)^64^; postprocessing was performed in MATLAB. Phase relations and rock properties were calculated between 4 and 16 kbar and 600 and 1,200 °C.

Bulk compositions for phase equilibria calculations are presented in Extended Data Table 1. For the crustal compositions (high-Si and low-Si; Extended Data Fig. 1), *x*Fe^3+^ (*x*Fe^3+^ = Fe^3+^/(Fe^2+^ + Fe^3+^)) was set to 0.2 on the basis of values reported for the enriched Archaean tholeiite (EAT) composition of ref. ^65^ in agreement with the general assumption that the Archaean surface environment was less oxygenated than modern environments of hydrothermal alteration for which *x*Fe^3+^ typically exceeds around 0.3 (for example, ref. ^66^). For sedimentary compositions, Fe_2_O_3_ was set to 0.1 mol% and we assume melting occurs under fluid-saturated conditions. Accordingly, bulk-rock H_2_O concentrations were adjusted so that minimal free H_2_O was present at the solidus at 10 kbar; this approach was adopted to ensure that melting initiated at the fluid-saturated solidus across the *P*–*T* range of interest.

All calculations were performed in the Na_2_O-CaO-K_2_O-FeO-MgO-SiO_2_-H_2_O-TiO_2_-O (NCKFMASHTO) subsystem. The effect of Mn on phase relations was not considered because of low concentrations of MnO in most of the compositions and uncertainties on the energetics of Mn-mixing between relevant mineral phases. Furthermore, the principal consequence of Mn on phase relations is to stabilize garnet to lower-grade *P–T* conditions^67^; such effects are expected to have a minor impact on supra-solidus phase relations. For silica-rich compositions (high-Si, siliciclastic, shale and Archaean shale; Extended Data Table 1) the following activity–composition models were used: silicate melt^68,69^; plagioclase feldspar^70^; epidote^64^; chlorite, chloritoid, biotite, garnet and orthopyroxene^68^; white mica^68,71^, magnetite and ilmenite^72^. For mafic compositions (low-Si and mafic sediment; Extended Data Table 1) we used the following activity–composition formulations: mafic melt, amphibole and clinopyroxene^73^; chlorite, garnet and orthopyroxene^68^; plagioclase^70^; olivine^64^; spinel^74^; magnetite and ilmenite^72^; epidote^64^; white mica^68,71^. The following pure phases were considered in all calculations: H_2_O, albite, quartz, kyanite, sillimanite, rutile, sphene, clinozoisite and zoisite.

**Extended Data Fig. 3:**
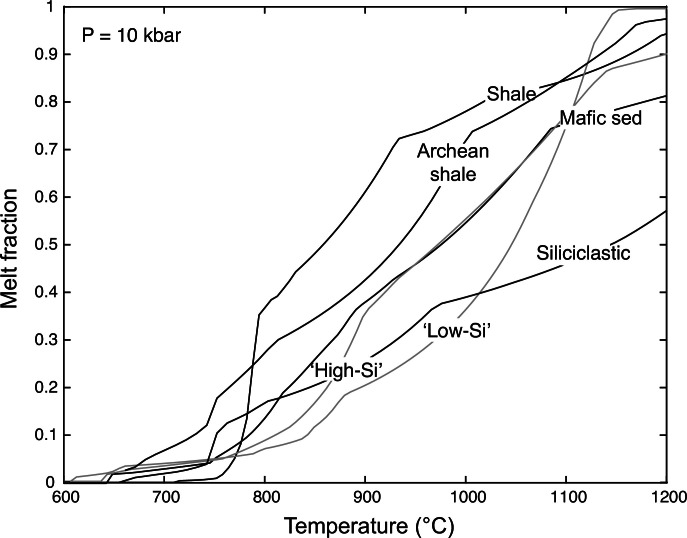
Variation of melt fraction (X) with temperature. Curves show calculated melt fractions plotted against temperature at 10 kbar for all compositions considered. See text for discussion.

Extended Data Fig. 3 shows the variation of melt fraction with temperature at 10 kbar for all bulk compositions considered. Assuming minimal saturation at the wet solidus, all compositions yield solidus temperatures between about 620 and 720 °C but melt fractions diverge at more than 700 °C between fertile shales and the remaining rock types. Curves for both shale compositions are characterized by abrupt increases in d*X*/d*T* less than 800 °C, caused by muscovite dehydration melting reactions; our exemplar Archaean shale composition is predicted to yield a melt fraction of about 0.3 by around 800 °C. Such melt fertility is in contrast to the remaining sediment compositions, as well as the high- and low-Si bulk compositions, which are characterized by melt fractions less than 0.2 at less than 800 °C. The broadly basaltic low-Si composition is predicted to yield large volumes of melt at elevated temperatures, between around 1,000 and 1,150 °C, whereas the TTG-like high-Si bulk produces most melt at slightly lower temperatures and over a greater temperature range, from around 850–1,100 °C. Note that the mafic sediment and high-Si compositions yield broadly similar melting curves at more than 900 °C. Finally, the siliciclastic composition is the least fertile bulk composition considered, yielding a melt fraction of only 0.6 at 1,200 °C because of the predominance of quartz in all phase assemblages.

**Extended Data Fig. 4:**
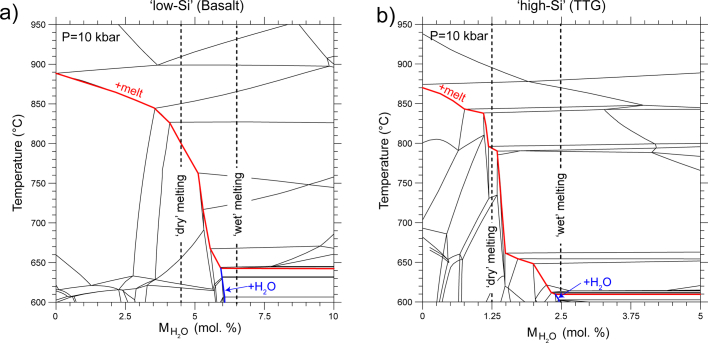
Effect of bulk-rock water concentration on solidus temperature for ‘low-Si’ and ‘high-Si’ compositions. Panel **a** shows T–X pseudosection calculated for ‘low-Si’ bulk composition; abscissa is bulk-rock molar % H_2_O. Red line represents the solidus and blue line represents the phase stability limit of free H_2_O. Thin lines correspond to mineral stability fields; labels omitted for clarity. Vertical dashed lines correspond to molar H_2_O concentrations used for ‘dry’ and ‘wet’ melting scenarios. Panel **b** is as for A except for the ‘high-Si’ composition; both panels calculated at 10 kbar.

To examine the effect of variable H_2_O concentrations on melt fertility, we constructed $T\mbox{--}{X}_{{{\rm{H}}}_{2}{\rm{O}}}$ curves for the low-Si and high-Si compositions. Solidus temperatures for both compositions range from less than 650 °C to more than 850 °C for saturated and ‘dry’ melting scenarios, respectively. For ‘wet’ melting, we used H_2_O concentrations that minimally saturate the system at 10 kbar (wet melting lines, Extended Data Fig. 4); for dry melting we adopted values of H_2_O that resulted in solidus temperatures around 800 °C. Reduced H_2_O concentrations would result in the onset of melting at higher temperatures.

We note that these *T–X* relations and melting curves are the result of continuous and discontinuous reactions amongst mineral phases and silicate melt. Interested readers are referred to refs. ^68,75^ for a detailed discussion of the phase relations of pelitic and TTG compositions at supra-solidus conditions, respectively.

### Thermal modelling

We model conduction and radiogenic heat production along a vertical column through the lithosphere^24,76–79^. The transient thermal field is given by:

1 $$\rho \frac{\partial {[C}_{p}\left(T\right)T+{LX}]}{\partial t}=\frac{\partial }{\partial z}\left[k(T)\frac{\partial T}{\partial z}\right]+A(z,t)$$

where $T$ is temperature, *t* is time, *ρ* is density, *C*_*p*_ is heat capacity, *k* is thermal conductivity, *L* is the latent heat of melting, *X* is melt fraction and *A* is radiogenic heat productivity. The effects of advection (erosion) and heat of sub-solidus metamorphic reaction on the thermal field were not considered. We use temperature-dependent values of thermal conductivity and heat capacity as this exerts an important insulating effect on the thermal evolution of the deep crust^80^. For crustal depths, we use the parameterization of ref. ^81^, whereas that of ref. ^82^ is used for the lithospheric mantle. The energetics of melt production were simulated assuming a value of 320 kJ kg^−1^ for *L* and the lithology-specific *T–X* parameterizations derived above. Values of 2,800 and 3,300 kg m^−3^ were assumed for crustal and mantle density, respectively. Crustal heat production was varied between model runs but all calculations used 0.006 μW m^−3^ for heat production in the mantle lithosphere^83^. The upper surface of the model domain was held at 0 °C for all times and the base of the model domain was held at constant temperature, defined by the initial geotherm and assumed lithospheric thickness. The initial (prethickening) geotherm was calculated using a steady-state formulation for a layered lithosphere with crustal and mantle heat production of 1.4 and 0.006 μW m^−3^, respectively, and a Moho heat flux of 13 mW m^−2^ (ref. ^84^). We assumed a constant thermal conductivity of 1.8 W m^−1^ K^−1^ for the initial crustal geotherm to avoid unreasonably cold geotherms calculated with the temperature-dependent conductivity model of ref. ^81^. Equation (1) was numerically integrated using an explicit finite difference scheme.

**Extended Data Fig. 5:**
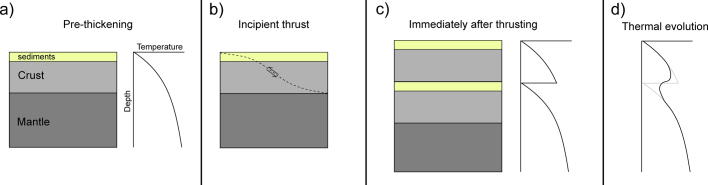
Physical sketch of thermal model. Prior to thickening the crust and mantle are in thermal equilibrium (**a**). Subsequent crustal thickening occurs along a crustal-scale thrust fault (**b**) and is approximated by instantaneous emplacement of a thrust sheet onto a continental section (**c**). The thermal structure then evolves via conductive relaxation of isotherms and radiogenic heating (**d**). Note that burial of HPE-enriched sediments (yellow layer) by this mechanism results in a different equilibrium thermal structure to that prior to deformation.

We assume that crustal thickening occurs by instantaneous emplacement of a single thrust sheet of variable thickness onto a continental section comprising crust and mantle lithosphere (Extended Data Fig. 5). This configuration of thickening is similar to that observed along modern convergent margins—for example, the Himalaya^85^ and Andes (for example, ref. ^86^; see ref. ^77^ for further details and examples)—in which low-grade rocks, including sediments, are delivered to middle- and lower-crustal depths along contractional faults. In our calculations, we consider the thermal effect of underthrusting sediment layers of variable thickness and heat production (Extended Data Figs. 9 and 10). Following emplacement of the thrust sheet, the initial ‘saw tooth’-shaped geotherm evolves in response to conduction and radiogenic heating. We do not consider the effects of heating during crustal thickening, nor the impact of a time-dependent mantle heat flux.

### Enrichment of Archaean/Palaeoproterozoic sediments in K and U

Radiogenic heat production in sedimentary rocks is controlled by depositional age and radioelement concentration at the time of sediment deposition. For a sediment of an average modern composition, the former parameter imposes an approximate increase of heat production by a factor of around 2 (at 2.8 Ga) relative to the modern sediment.

**Extended Data Fig. 6:**
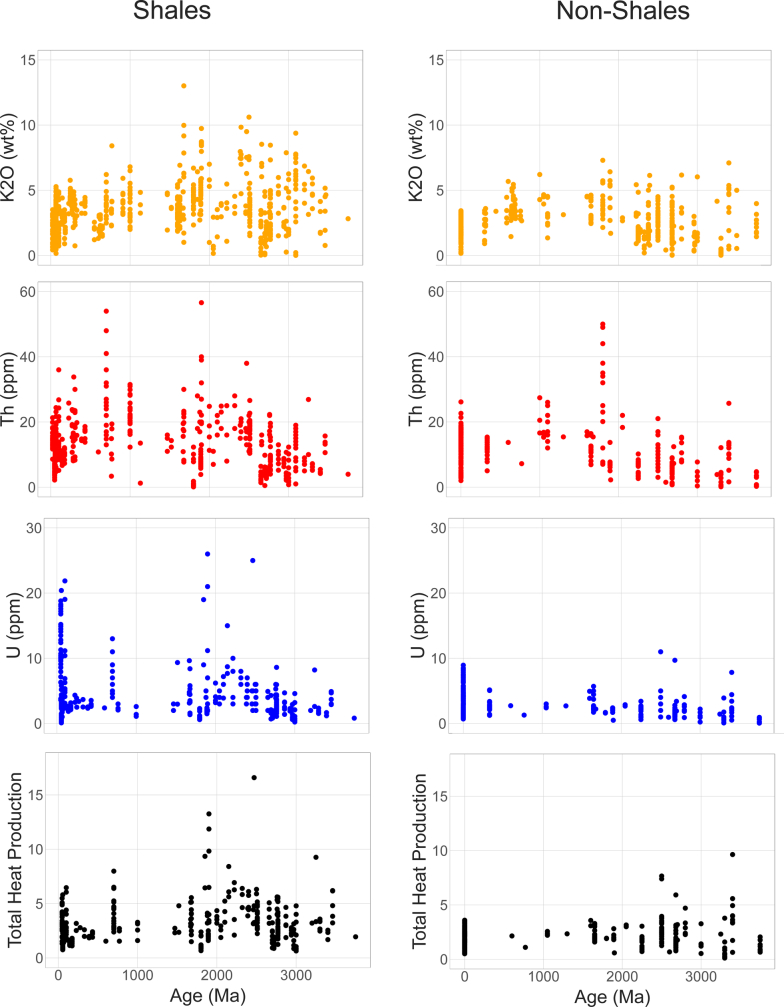
Secular variations in concentrations of radioelements, K, Th and U, in shales and non-shale sediments. Lowermost panels show volumetric heat production calculated at the age of sediment deposition. Data are from the global compilation of ref. ^30^.

**Extended Data Fig. 7:**
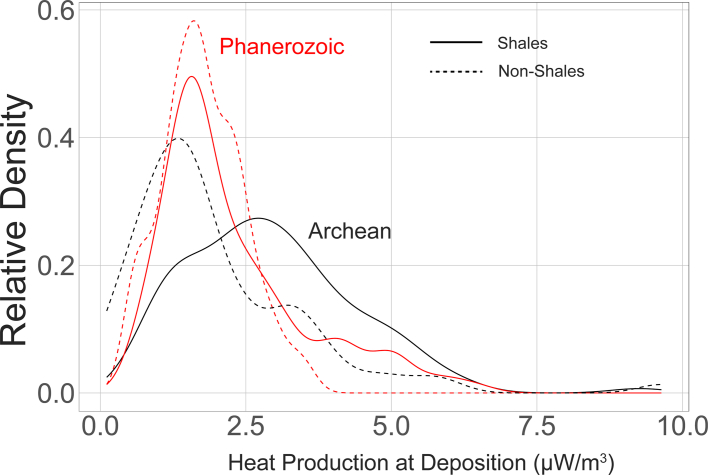
Probability density plots of radiogenic heat production for Archean and Phanerozoic sediments. Shales are plotted as solid lines, whereas ‘non-shale’ clastic sediments are plotted as dashed lines. Data are from the global compilation of ref. ^30^.

Extended Data Fig. 6 shows measured concentrations of U, Th and K_2_O plotted against the age of sediment deposition for shales and ‘non-shale’ sediment compositions, as defined in the compilation of ref. ^30^. The figure shows that K_2_O concentrations in all clastic sediments broadly decrease from peak values at around 2 Ga; U concentrations in shales seem to follow a similar trend, although modern black shale concentrations span a large range of concentrations up to 20 ppm. Thorium concentrations in clastic sediments increased from around 3–2 Ga, after which the data are highly dispersed. A consequence of these secular variations in radioelement concentrations is that shales deposited between 2 and 2.5 Ga have elevated heat production relative to Phanerozoic shale compositions (Extended Data Fig. 7—the median heat production for Phanerozoic shales is 1.81 μW m^−3^ (*Q*_1_ = 1.47 μW m^−3^, *Q*_3_ = 2.8 μW m^−3^) compared to 2.76 μW m^−3^ for Archaean shale (*Q*_1_ = 1.85 μW m^−3^, *Q*_3_ = 3.75 μW m^−3^)).

Uranium enrichment in Neoarchaean/Palaeoproterozoic shales is broadly contemporaneous with oxidation of the atmosphere^87,88^. The onset of oxidative weathering is expected to have solubilized U as mobile U(VI) complexes^89^, increasing the continental U flux to marine basins^90^. Before about 2.5 Ga, U was probably immobilized as U(IV) in plagioclase in exposed granitoids and detrital mineral phases, such as uraninite, pyrite and siderite, in shallow-marine sediments^91,92^.

### Geology of Archaean cratons

Here, we provide salient details of the geological histories of several Archaean cratons for which our proposed model provides a viable mechanism to explain Neoarchaean intracrustal melting and granitoid formation.

#### The Slave craton

This craton contains some of the most expansive Archaean sedimentary sequences known globally. The general geology has been summarized by many previous workers^7,42,93–95^. The Neoarchaean record is defined by a thick package of tholeiitic submarine volcanic sequences (the Kam group) that erupted onto basement gneisses and thin packages of cover-group sandstones, banded iron formations and conglomerates. The mafic volcanic rocks generated an extrusive package of 1–6 km thickness that was succeeded by a phase of calc-alkaline volcanism. The predominant sedimentary rock sequences comprise two distinct packages of turbidites that were deposited between 2.66 and 2.61 Ga (ref. ^93^). Limited geochemical analyses exist for these rocks but in some areas the packages consist of more than 5 km of interbedded sandstones, siltstones and black slates^96^. Lower-crustal xenolith suites, overwhelmingly associated with the Lac de Gras kimberlite field, Eastern Slave, are dominated by mafic granulites but contain populations of metasedimentary granulites^34,42,97^. These granulite xenoliths record peak pressures of 0.8–1.2 GPa, indicating that they were sourced from Moho depths during the kimberlite volcanism of 55 million years ago (Ma) that brought them to the surface. Metasedimentary granulites have refractory compositions, indicating that they have undergone substantial melt loss; they have heat productivities of around 0.29 μW m^−3^ (ref. ^34^). Metamorphic zircon growth occurred in Slave craton xenoliths in several intervals between 2.64 and 2.51 Ga (ref. ^42^), overlapping with the timing of plutonism represented by the 2.62–2.58 Ga granite-bloom event in the Slave province. Thus, it is plausible that Neoarchaean plutonism in the Slave province was driven by heat production in response to the addition of sedimentary materials into the lower crust during Neoarchaean assembly of the craton.

#### The Superior craton

This craton is the largest, best-exposed and most intensely studied of the Archaean cratons. The craton has been subdivided into east–west trending provinces that are commonly fault-bounded, defining a lateral structure which has been used to argue for accretionary orogenic processes that sutured the Superior province into its present configuration^98^. A period of (ultra-)high-temperature metamorphism is recorded in the very large Pikwitonei granulite terrane at 2.68 Ga (ref. ^99^), which contains metre- to kilometre-scale bands or rafts of metasedimentary protoliths, although most of the terrane is dominated by meta-igneous rocks. The adjacent North Caribou terrane contains a history of sedimentation dating back to about 3.0 Ga (ref. ^100^), although classic wedge deposits are not typically found until 2.7 Ga. Across other parts of the craton, sedimentation occurred between 2.72 and 2.68 Ga, whereas granitoid magmatism lags by 20–40 Ma. This typically coincides with metamorphism where preserved^101^.

#### The Amazonian craton

This craton is formed by two Archaean nuclei (the Guiana and Guaporé shields), separated by the Amazonian cratonic basin. Across the craton, TTG magmatism occurred between around 3 and 2.89 Ga and was succeeded by emplacement of sanukitoids and anatectic granites until approximately 2.84 Ga. In the Carajás province, emplacement of these anatectic granites was associated with crustal thickening and granulite-facies metamorphism^102^.

#### The Pilbara craton

This craton is the classic granite-greenstone cratonic structure with domal granitic provinces intruding into older basaltic supracrustal packages. The preservation of the Pilbara craton is exceptional, with limited deformation and erosion as compared to other cratons—thereby providing an excellent window into surficial evolution but a poorer record of metamorphism in the deep crust. The Palaeoarchaean/Mesoarchaean history of the Pilbara is dominated by plume-vertical processes^103,104^ that constructed the East Pilbara terrane. Up to 9–18 km of stratigraphy was developed by a combination of igneous and sedimentary rocks. Significant shale deposition does not occur until around 3.0 Ga (ref. ^105^) and no high-grade metamorphic sequences are recorded in the Pilbara, apart from contact metamorphism surrounding late granite plutons^104^

#### The Karelian craton

This craton spans about 400,000 km^2^ of the Baltic shield (northeastern Finland and adjacent Russia) and is dominated by late Archaean TTG gneisses, greenstone belts, diorite-to-granite plutons and migmatitic metasediments. Emplacement of TTG granitoids occurred between around 2.95 and 2.75 Ga before a phase of sanukitoid magmatism, culminating in biotite and two-mica granite emplacement between 2.75 and about 2.63 Ma (see reviews by refs. ^2,106^). Deposition of wackes and shales that form the protoliths of amphibolite-grade paragneisses occurred between 2.71 and 2.69 Ma, swiftly followed by regional amphibolite- and granulite-facies metamorphism from 2.7 to 2.63 Ga (refs. ^107,108^).

#### The Kaapvaal craton

This craton comprises an older-joined crust and mantle root compared to the Slave craton as indicated by the geochronological investigations that constrain the last Archaean magmatic event to around 3.1 Ga (ref. ^109^), apparently due to intracrustal melting during amalgamation of Mesoarchaean continental blocks. After this time, the continental block or tectosphere, was stable and provided freeboard to erode and deposit large sedimentary basins such as the Witwatersrand Basin at 2.7 Ga. A large, near-complete, cross-section of Archaean Kaapvaal crust was exposed during the Vredefort impact event, 2.0 Ga (ref. ^110^). Important to the proposed hypothesis is the fact that a significant amount of the Mesoarchaean lower crust exposed by the Vredefort impact is metasedimentary. The exposed rocks include sapphirine-bearing granulites, interlayered felsic gneiss, felsic charnockites and paragneisses^35,43^. Detailed mapping and geochronology of this exposed lower crust provided evidence for large volumes (approximately 40%) of melting of pre-existing mafic and felsic lower crust in the craton around 3.1 Ga. Geochronological investigations constrain the age of crust-mantle coupling to between 3.09 and 3.07 Ga. However, ref. ^35^ argued that the degree of thermal reworking at 3.08 Ga was inconsistent with the presence of a deep, cool mantle root at that time on the grounds that a root would have served to impede heat transfer. Thus, the crust and mantle were probably aggregated independently. Our proposed mechanism, in which intracrustal melting is instead driven by radiogenic heat delivery to the lower crust by underthrusting of sedimentary rocks, provides a viable alternative to independent formation and aggregation of Archaean crust and mantle components. This model draws strong support from the Kaapvaal craton, for which there is clear evidence for large volumes of residual metasedimentary rocks being present in the lower crust before emplacement of granite intrusions at 3.1 Ga (refs. ^35,43^). Indeed, ref. ^44^ invoked a comparable model to explain zircon Hf- and O-isotope trends from 3.1 Ga peraluminous granitic rocks exposed in the Grunehogna craton in East Antarctica—interpreted to be a part of the Kaapvaal craton lithosphere in the Archaean period.

#### The Dharwar craton

This is the largest of the five cratonic shields that collectively form Peninsular India and comprises a western shield (3.3–2.7 Ga) and a younger eastern shield (3.0–2.5 Ga). The western shield is dominated by TTG gneisses overlain by greenstone belts; by contrast, greenstone belts are rare in the eastern shield, where late Archaean (2.6–2.5 Ga) granitoids intruded older TTG (2.9–2.7 Ga) gneisses (refs. ^111,112^ and references therein). Amalgamation of the eastern and western shields is constrained to 2.7 Ga. Supracrustal rocks of the Dharwar supergroup were deposited between 2.9 and 2.72 Ga on the basis of ages of metavolcanic horizons. Biotite and two-mica granites were emplaced between 2.54 and 2.51 Ga, contemporaneous with regional amphibolite- to granulite-facies metamorphism at 2.51–2.52 Ga (refs. ^113,114^).

#### The North China craton

This craton spans around 1.7 million km^2^ across northeastern China, Inner Mongolia and North Korea and is formed from a mosaic of microblocks that amalgamated before 2.5 Ga. Predominant lithologies vary considerably between microblocks but all rocks with ages more than 2.5 Ga are affected by Neoarchaean metamorphism and were intruded by 2.5–2.45 Ga granitic bodies. Across the craton, TTG plutonism occurred between 2.75 and 2.55 Ga; sanukitoids were emplaced in a short time period (2.55–2.52 Ga) that overlapped with emplacement of biotite and two-mica granites (2.55–2.44 Ga). Metapelitic granulites preserve evidence for the incorporation of near-surface rocks into the lower crust along clockwise pressure–temperature paths, culminating at peak conditions at 2.49 (Qingyuan terrane, eastern North China craton; for example, ref. ^115^) and around 2.52 Ga (Yinshan block, western North China craton; for example, ref. ^116^).

### Predictions about volumes of peraluminous melts

**Extended Data Fig. 8:**
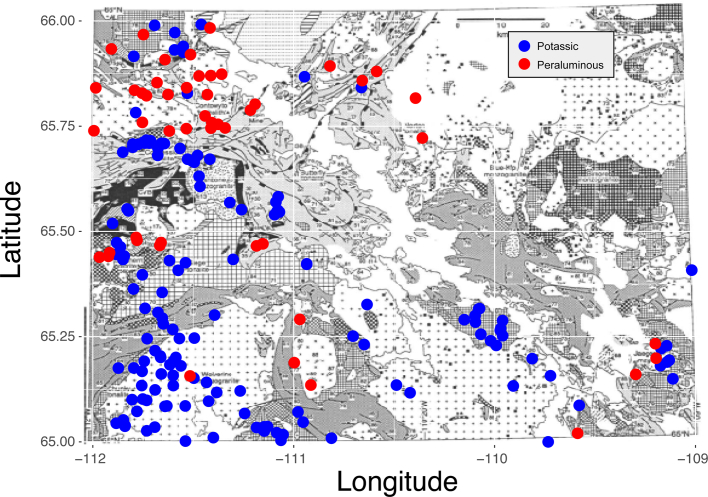
Map of a granitoid province in the NE Slave craton, after ref. ^16^. Data points are granitoid samples coloured according to their composition and source characteristics.

**Extended Data Fig. 9:**
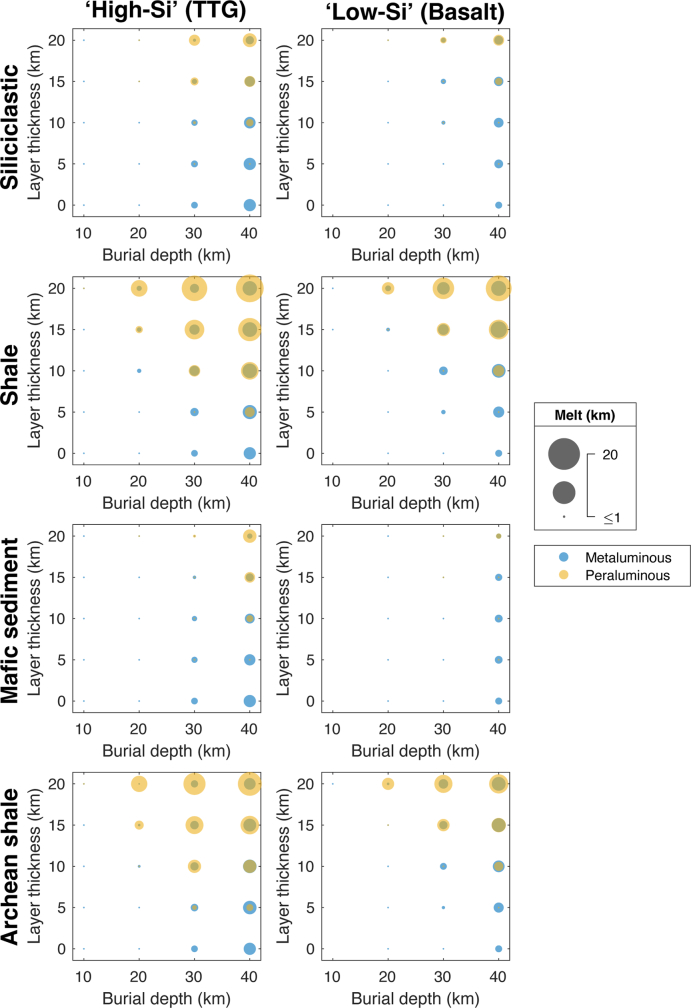
Quantities of melt produced from ‘hydrous’ melting of Archean crustal and sedimentary lithologies. Sub-plot columns represent the background composition of the crustal column (that is TTG and basalt), while the sub-plot rows represent the composition of sedimentary layers incorporated in the thermal models. For each individual sub-plot, the x-axis indicates the depth of burial for a given thickness of sedimentary layer, shown on the y-axis. Volumes of igneous (metaluminous) and sediment-derived (peraluminous) melt are represented by the radii of the blue and yellow circles, respectively.

In the mechanism proposed here, intracrustal differentiation and craton stabilization are facilitated by radiogenic heat produced in metasedimentary rocks. An obvious prediction is that peraluminous—that is, sedimentary—melts would be expected to dominate the Neoarchaean rock record. Compilations of Neoarchaean granitoids show, however, that such peraluminous granitoids are subordinate to I-type, metaluminous, melts across most cratons (for example, ref. ^15^). There are several factors that reconcile this observation with sediment-driven crustal differentiation:

1. Neoarchaean detrital zircons tend towards elevated δ^18^O values relative to those preserved by the magmatic rock record (ref. ^49^, their figure 1), consistent with the erosional removal of differentiated and, in our model, high-heat production peraluminous melts from the rock record.
2. There are cratonic locales where substantial volumes of peraluminous granites are locally preserved. Extended Data Fig. 8 shows superimposed granite samples (coloured symbols) onto a geological map^31^ from the Slave craton. Red data points represent rocks mapped as sediment-derived melts whereas the blue points are mapped as metaluminous or potassic granites. Clearly, a substantial volume of peraluminous granite is exposed in this large (more than 1,500 km^2^) region, consistent with constraints from seismic wavespeeds and metamorphic rock compositions (see main text).
3. Our calculations show that the incorporation of radioactive sedimentary material into the deep crust stimulates the production of melt from proximal—but non-metasedimentary—lithologies. Extended Data Fig. 9 shows the volumes of melt produced from metasedimentary (yellow circles) and TTG sources (blue circles) for various burial depths and metasedimentary layer thicknesses. Indeed, certain configurations predict a 1:1 ratio of metasedimentary and TTG melts (for example, 10 km of Archaean shale incorporated at 40 km depth). Owing to decreasing d*T*/d*z* with depth, the deeper a metasedimentary package is buried, the more potential there is for the stimulation of melt from proximal TTG crust. This mechanism would account for the observed dilution of pure S-type melts with I-type granitoids in Neoarchaean cratons. Furthermore, metasediment-derived melts generated in the deep crust will also be susceptible to assimilation, the generation of mixed melts on ascent and the potential erasure of their hallmark peraluminous nature.

### Further results from thermal modelling

**Extended Data Fig. 10:**
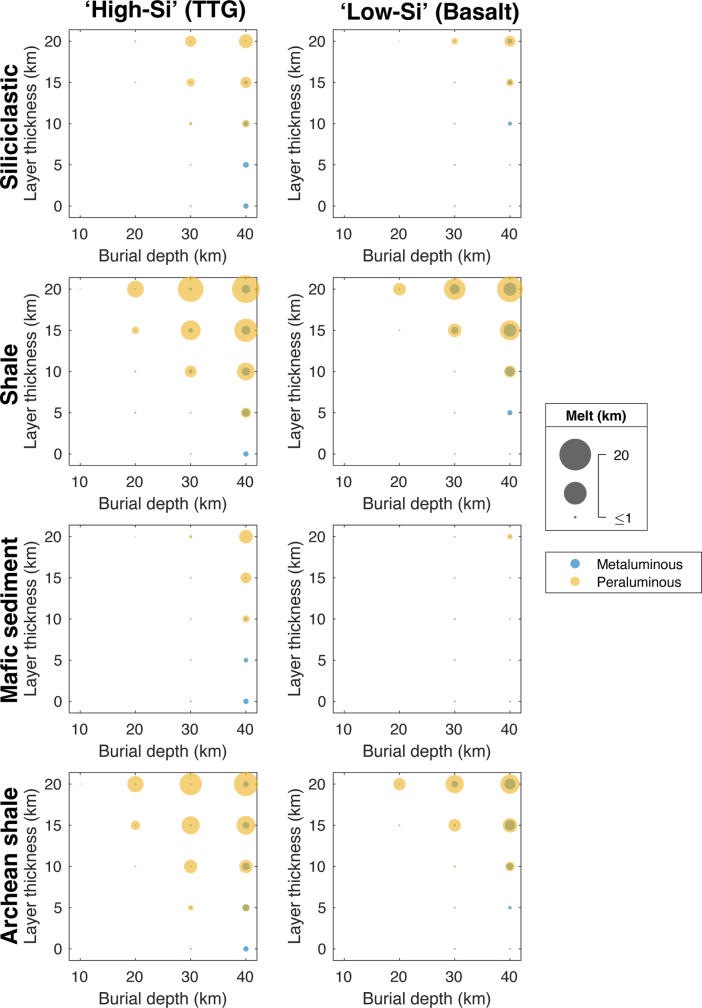
Quantities of melt produced from ‘dry’ melting of Archean crustal and sedimentary lithologies. Panel is as for Extended Data Fig. 9 except T–X relations for ‘high-Si’ and ‘low-Si’ compositions are calculated using ‘dry’ bulk compositions.

Extended Data Figs. 9 and 10 show the quantities of sediment-derived (S-type or peraluminous) and crustal-derived (I-type or metaluminous) granitoid melt produced for various combinations of crustal hydration state, sediment composition, layer thickness and burial depth. Melt quantities are calculated by vertical integration of the melting column (for example, Fig. 3) and are expressed as circular markers with radii scaled to the total km of melt produced after 50 Myr. Visual inspection of these results shows that thickening of pure-TTG or basaltic crust (corresponding to layer thicknesses of 0 km) produces minimal melt (less than 4 km for wet melting, Extended Data Fig. 9; less than 1.5 km for dry melting, Extended Data Fig. 10) and such melting is restricted to depths of more than 30 km. Shales (shale and Archaean shale compositions) are the most fertile of the sediment compositions due to mica-rich phase relations that result in characteristic steep d*T*/d*X* melting curves (Extended Data Fig. 5) at temperatures proximal to the wet solidus. By contrast, burial of mafic sediment produces the smallest melt fractions as a combined result of shallow d*T*/d*X* melting curves at less than 800 °C (Extended Data Fig. 4) and low radiogenic heat production (Fig. 2 and Extended Data Fig. 2).

Extended Data Figs. 9 and 10 also show that burial of shales into the deep crust stimulates the production of granitoid melts from adjacent TTG and basaltic rocks. For example, crustal thickening of 30 km of dry TTG (high-Si composition) results in around 0.3 km of I-type granitoid melt (metaluminous) in comparison to around 1 km when 15 km of Archaean shale (3.4 μW m^−3^) is buried to 30 km. Stimulation of such melt occurs predominantly in response to downward conduction of radiogenic heat produced in the sediment layer—an effect that could plausibly lead to melt production in the lithospheric mantle beneath orogens. This effect is restricted to shale compositions; burial of both the siliciclastic and mafic sediment compositions does not stimulate melting of adjacent TTG (Extended Data Fig. 9, left column, first and third rows), as these sediments do not have heat production rates that are significantly elevated over the host TTG composition.

## Online content

Any methods, additional references, Nature Portfolio reporting summaries, source data, extended data, supplementary information, acknowledgements, peer review information; details of author contributions and competing interests; and statements of data and code availability are available at 10.1038/s41586-024-07307-1.

## Figures and Tables

**Extended Data Table 1:**
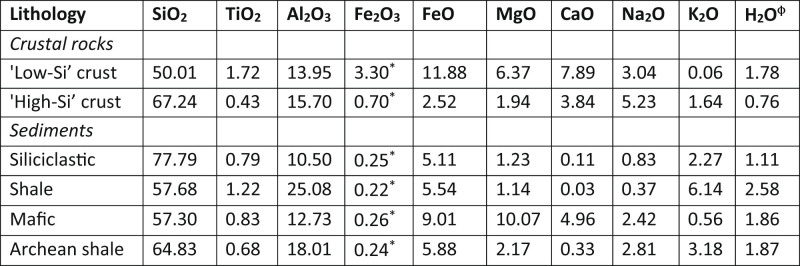
Bulk-rock compositions used for phase equilibria calculations (weight % oxides)

Definition of suprascript symbols: ^Ψ^Fe_2_O_3_ set to 0.1 mol.%; ^*^ XFe^3+^ set to 0.2, where XFe^3+^ = (2(Fe_2_O_3_)/(FeO + 2(Fe_2_O_3_)); ^ϕ^H_2_O concentration required for minimal saturation at the solidus, P *=* 10 kbar. ‘Archean shale’ composition derived from shales with depositional ages >2.5 Ga, whereas ‘Shale’ composition include rocks of all ages.

## Data Availability

The data that support the findings of this study are available from the corresponding author upon reasonable request.

## Code availability

Computer codes replicating the results in this study can be obtained from the corresponding author on request.
